# COVID-19: an FY1 on the frontline

**DOI:** 10.1080/10872981.2020.1759869

**Published:** 2020-04-28

**Authors:** Priyancaa Jeyabaladevan

**Affiliations:** Foundation Doctor, Barts NHS Trust, London, UK

**Keywords:** COVID-19, coronavirus, junior doctor, foundation training, redployment

## Abstract

The global spread of COVID-19 has put increased pressure on the NHS. The Government has put in a number of strategies to cope with this pandemic, which includes increasing funding for the NHS. However, increased funding itself will not ease the workload. With a number of our staff isolating from work due to COVID-19, we as the workforce have to step out of comfort zones and work in unfamiliar specialties. These are unprecedented times and are placing strains on our health service. Nonetheless, we as healthcare professionals have taken oaths that we are honouring and will continue to do so, till this virus is put to rest.

I am a General Surgical FY1 at a district general hospital in London. When COVID–19 hit the UK, my colleagues and I knew we would need to step up and adapt to prepare for this global pandemic. When I graduated medical school, never did I think that within seven months of becoming a doctor, I would be working in such a climate. Over the past few weeks, I have witnessed difficult decisions being made and difficult conversations being had. I have heard doctors telling families over the phone that their loved ones, who they have not been able to see because of risk of exposure, were dying and that they did not have long left to live. The four pillars of medical ethics [1] were drilled into us at medical school, but now more than ever do we as healthcare professionals find ourselves thinking about it more and more to ensure that we do right by our patients to maintain their autonomy in these trying times.

As foundation year trainees, we were meant to move onto the next rotation on the 1^st^ of April 2020. With increased demands of doctors needed to be covering the wards, Health Education England [2] decided that we would not receive adequate induction and we should remain in our current placement till informed otherwise.

Pre–COVID pandemic, a General Surgery on–call would amass on average around forty inpatients over four days. However, the last two on–calls, we have only admitted on average ten patients. I think this is because patients understandably do not want to present to hospital as they fear becoming infected by COVID. Nevertheless, the incidences of common surgical presentations, such as appendicitis and abscesses has decreased. This brings the issue of patients in the coming weeks being admitted with delayed presentations, leading to worse outcomes. With the reduced patient load, as surgical juniors, we are more available to help our medical colleagues. What I have appreciated most in these unprecedented times, is how we have banded together to deal with this emergency. It is heart-warming to see radiologists, haematologists and ophthalmologists and many more volunteer themselves to come back and work on the wards as juniors to help ease the pressure on our medical counterparts.

To ensure our staff are working effectively, the department leads have acted quickly to devise an emergency COVID rota. The surgical team has now reduced to a skeletal team, which covers the on-call and existing inpatients. The remaining surgical staff are deployed to the critical care team to staff the supplementary ICU. I worked my first weekend shift on the ICU on 28^th^ – 29^th^ of March. Initially, I was anxious as I would be working in a new environment with critically ill COVID patients, hooked up to machines I was unfamiliar with. However, I quickly eased into the role as my team were extremely supportive. There were two anaesthetic consultants leading the team who were appreciative of having extra bodies and were fully understanding of my inexperience in this setting. They took their time to show me where I could find appropriate PPE and emphasised the importance of proper safety procedures. During this shift was the first time that I had worn full PPE for an extended period of time. The ward round only lasted about forty-five minutes but wearing the respirator mask for this long was challenging. It was difficult for us to communicate with each other as our voices were muffled by the mask. At the end of the shift, I found myself more tired than usual. Donning and doffing full PPE is necessary and important but also exhausting. It makes simple clinical skills such as a catheter change more laborious. I came to have a far greater appreciation for ICU nurses. I found it challenging simply wearing full PPE for ward round, yet they would spend the majority of their shift in the full PPE caring for the patient.

COVID-19 has been frightening. The future is uncertain and we as healthcare professionals have stepped out of our comfort zones. Despite all of this, from what I have seen, we have absolutely risen to the occasion. I am grateful for how well Trusts are dealing with this pandemic – not only in looking after patients but also in looking after its staff. The public have been very generous in their donations to organisations such as ‘Meals for the NHS’, who are providing us with a constant supply of hot and delicious food. Though our service is being stretched, from my first-hand experience, I can wholly say that we are successfully persevering in upholding NHS’ core value of delivering the best patient centred care we can.
